# Supplementary material for: MetaRibo-Seq measures translation in microbiomes
Source: Nat Commun. 2020 Jun 29;11:3268. doi: 10.1038/s41467-020-17081-z (PMC7324362; doi:10.1038/s41467-020-17081-z)
Supplement: Supplementary file 10 — Supplementary Data 7 [file 41467_2020_17081_MOESM10_ESM.zip › File2/Confidence_High_Taxonomy/380793_out.krona.html]

Javascript must be enabled to view this page.

members
magnitude
magnitudeUnassigned
count
unassigned
taxon
rank

380793\_out

5

2
superkingdom
5

5
1239
phylum

class
186801
5

5
order
186802

186803
family
2

2
2569097
genus

species
39488

SRS019068\_contig\_number\_82834SRS050422\_contig\_number\_14426
2

3
186806
family

3

SRS053214\_contig\_number\_11039SRS147346\_contig\_number\_contig-100\_22641.22641SRS148159\_contig\_number\_50544
1730
genus
